# Finding hotspots: the role of active surveillance methods in malaria control and elimination

**DOI:** 10.1186/1475-2875-11-S1-O6

**Published:** 2012-10-15

**Authors:** Hugh JW Sturrock, Teun Bousema, Jacklin Mosha, Roly D Gosling

**Affiliations:** 1 Malaria Elimination Initiative, Global Health Group, University of California San Francisco, CA 94105, USA; 2 Department of Immunity and Infection, London School of Hygiene & Tropical Medicine, London, WC1E7HT, UK; 3 Kilimanjaro Christian Medical College, Moshi, Tanzania

## 

It is evident that malaria infection and transmission display fine scale clustering over all transmission settings. Such clusters, or hotspots, could be a group of households, or even a single household, whose occupants suffer from an abnormally high exposure to infectious mosquitoes and are a source of infection to households outside the cluster. Whilst it has been suggested that targeting interventions at hotspots is likely to be a cost-effective method to reduce transmission, the challenge remains to develop methods for their identification. Active Case Detection (ACD), whereby defined populations are screened and treated where necessary, may offer one solution. There are, however, a number of factors that need to be considered before ACD is implemented, including its timing and frequency, whether it should be conducted pro- or re-actively, transmission setting and diagnostic method used. This presentation will examine and discuss the potential use and effectiveness of ACD in relation to the spatial epidemiology of malaria using examples over different transmission settings.

